# Supplementary material for: Short-Term Panax Ginseng Extract Supplementation Reduces Fasting Blood Triacylglycerides and Oxygen Consumption during Sub-Maximal Aerobic Exercise in Male Recreational Athletes
Source: Biomolecules. 2024 Apr 30;14(5):533. doi: 10.3390/biom14050533 (PMC11118118; doi:10.3390/biom14050533)
Supplement: Supplementary file 1 [file biomolecules-14-00533-s001.zip › biomolecules-2949612-supplementary.pdf]

Supplementary files

Short-term Panax Ginseng extract supplementation reduces fasting blood triacylglycerides and oxygen consumption during sub-maximal aerobic exercise in male recreational athletes.

Didier Hernández-García[1], Ana Belén Granado-Serrano[1], Meritxell Martín-Gari[1], Assumpta Ensenyat[2], Alba Naudí[1], and Jose CE Serrano[1,*]

[1] NUTREN-Nutrigenomics, Department of Experimental Medicine, Universitat de Lleida, Lleida, Spain
[2] Institut Nacional d'Educació Física de Catalunya, Lleida, Spain.
* Correspondence: josecarlos.serrano@udl.cat; Tel.: +34 973702408

Table S1. Two-way ANOVA statistical analysis results for treatment effect of volunteer's demographic, training, and nutritional parameters.

Parameter	F	p-value	Partial eta squared
**A. 10 km races**			
Body weight	1.083	0.3025	0.0183
Training parameters			
Km/week	6.569	0.0130	0.1017
min/week	1.948	0.1681	0.0325
Nutritional parameters			
Energy	1.976	0.1652	0.0329
Carbohydrates	14.17	0.0004	0.1963
Protein	1.061	0.3072	0.0180
Lipids	0.667	0.4176	0.0114
**B. Sub-maximal performance test**			
Body weight	3.503	0.0694	0.0884
Training parameters			
Km/week	6.502	0.0152	0.1529
min/week	0.007	0.9353	0.0002
Nutritional parameters			
Energy	5.217	0.0284	0.1266
Carbohydrates	4.682	0.0372	0.1151
Protein	3.679	0.0631	0.0927
Lipids	0.712	0.4042	0.0194

Table S2. Two-way ANOVA statistical analysis results for time, blood and perceived effort parameters of volunteer's 10 km races.

Parameter	F	p-value	Partial eta squared
**A. Race time**			
Time (1st race vs 2nd race)	33.87	<0.0001	0.5387
Treatment (Placebo vs PG)	1.721	0.1999	0.7853
Multiple comparison			
Placebo (1st race vs 2nd race)		<0.0001	
Panax ginseng (1st race vs 2nd race)		0.0020	
First race (Placebo vs PG)		0.1615	
Second race (Placebo vs PG)		0.2412	
**B. Borg's CR-10**			
Time (1st race vs 2nd race)	4.899	0.0349	0.1445
Treatment (Placebo vs PG)	0.5630	0.4591	0.0680
Multiple comparison			
Placebo (1st race vs 2nd race)		0.6459	
Panax ginseng (1st race vs 2nd race)		0.3870	
First race (Placebo vs PG)		0.2142	
Second race (Placebo vs PG)		0.0743	
**C. Total lipids**			
Before 10 km race			
Time (1st race vs 2nd race)	0.0823	0.7745	0.0029
Treatment (Placebo vs PG)	2.033	0.1646	0.3289
Multiple comparison			
Placebo (1st race vs 2nd race)		0.0412	
Panax ginseng (1st race vs 2nd race)		0.0196	
First race (Placebo vs PG)		0.8580	
Second race (Placebo vs PG)		0.0158	
After 10 km race			
Time (1st race vs 2nd race)	2.622	0.1162	0.0829
Treatment (Placebo vs PG)	0.1195	0.7321	0.0315
Multiple comparison			
Placebo (1st race vs 2nd race)		0.2610	
Panax ginseng (1st race vs 2nd race)		0.2621	
First race (Placebo vs PG)		0.7520	
Second race (Placebo vs PG)		0.7398	
Placebo			
Time (Pre – post race)	30.21	<0.0001	0.5017
10 km race (1st vs 2nd race)	0.8925	0.3523	0.1551
Multiple comparison			
Pre-race (1st race vs 2nd race)		0.4713	
Post-race (1st race vs 2nd race)		0.7703	
Pre-post race (1st race)		0.0003	
Pre-post race (2nd race)		0.0034	
Panax ginseng			
Time (Pre – post race)	72.03	<0.0001	0.7201
10 km race (1st vs 2nd race)	0.0772	0.7832	0.0184
Multiple comparison			

Pre-race (1st race vs 2nd race)		0.3005	
Post-race (1st race vs 2nd race)		0.6005	
Pre-post race (1st race)		0.0001	
Pre-post race (2nd race)		<0.0001	
**D. Phospholipids**			
Before 10 km race			
Time (1st race vs 2nd race)	2.300	0.1348	0.0381
Treatment (Placebo vs PG)	12.52	0.0008	0.1776
Multiple comparison			
Placebo (1st race vs 2nd race)		<0.0001	
Panax ginseng (1st race vs 2nd race)		0.4775	
First race (Placebo vs PG)		0.0333	
Second race (Placebo vs PG)		<0.0001	
After 10 km race			
Time (1st race vs 2nd race)	0.1702	0.6814	0.0029
Treatment (Placebo vs PG)	2.289	0.1357	0.0380
Multiple comparison			
Placebo (1st race vs 2nd race)		0.4322	
Panax ginseng (1st race vs 2nd race)		0.1854	
First race (Placebo vs PG)		0.0366	
Second race (Placebo vs PG)		>0.9999	
Placebo			
Time (Pre – post race)	6.093	0.0164	0.0922
10 km race (1st vs 2nd race)	0.3245	0.5711	0.0054
Multiple comparison			
Pre-race (1st race vs 2nd race)		0.2871	
Post-race (1st race vs 2nd race)		0.7892	
Pre-post race (1st race)		0.0187	
Pre-post race (2nd race)		0.2871	
Panax ginseng			
Time (Pre – post race)	10.00	0.0025	0.1516
10 km race (1st vs 2nd race)	0.7844	0.3796	0.0138
Multiple comparison			
Pre-race (1st race vs 2nd race)		0.1129	
Post-race (1st race vs 2nd race)		0.7218	
Pre-post race (1st race)		0.0021	
Pre-post race (2nd race)		0.2156	
**E. Total cholesterol**			
Before 10 km race			
Time (1st race vs 2nd race)	0.0435	0.8362	0.0015
Treatment (Placebo vs PG)	1.850	0.1843	0.2214
Multiple comparison			
Placebo (1st race vs 2nd race)		0.2374	
Panax ginseng (1st race vs 2nd race)		0.3874	
First race (Placebo vs PG)		0.5507	
Second race (Placebo vs PG)		0.0682	
After 10 km race			
Time (1st race vs 2nd race)	2.143	0.1540	0.0688
Treatment (Placebo vs PG)	0.6278	0.4346	0.1946

Multiple comparison			
Placebo (1st race vs 2nd race)		0.3014	
Panax ginseng (1st race vs 2nd race)		0.3167	
First race (Placebo vs PG)		0.4509	
Second race (Placebo vs PG)		0.4509	
Placebo			
Time (Pre – post race)	1.976	0.1701	0.0618
10 km race (1st vs 2nd race)	0.3672	0.5491	0.1056
Multiple comparison			
Pre-race (1st race vs 2nd race)		0.4619	
Post-race (1st race vs 2nd race)		0.6810	
Pre-post race (1st race)		0.5423	
Pre-post race (2nd race)		0.1804	
Panax ginseng			
Time (Pre – post race)	0.0756	0.7854	0.0027
10 km race (1st vs 2nd race)	0.0055	0.9411	0.0017
Multiple comparison			
Pre-race (1st race vs 2nd race)		0.5855	
Post-race (1st race vs 2nd race)		0.6853	
Pre-post race (1st race)		0.3950	
Pre-post race (2nd race)		0.2207	
**F. Triacylglycerides**			
Before 10 km race			
Time (1st race vs 2nd race)	0.0641	0.8018	0.0022
Treatment (Placebo vs PG)	5.452	0.0267	0.4193
Multiple comparison			
Placebo (1st race vs 2nd race)		0.8023	
Panax ginseng (1st race vs 2nd race)		0.5550	
First race (Placebo vs PG)		0.0763	
Second race (Placebo vs PG)		0.0219	
After 10 km race	4.257	0.0481	0.8098
Time (1st race vs 2nd race)	5.009	0.0331	0.9240
Treatment (Placebo vs PG)			
Multiple comparison			
Placebo (1st race vs 2nd race)		0.0350	
Panax ginseng (1st race vs 2nd race)		0.4713	
First race (Placebo vs PG)		0.0181	
Second race (Placebo vs PG)		0.1868	
Placebo			
Time (Pre – post race)	0.6121	0.4401	0.0200
10 km race (1st vs 2nd race)	0.5984	0.4453	0.0834
Multiple comparison			
Pre-race (1st race vs 2nd race)		0.9041	
Post-race (1st race vs 2nd race)		0.1332	
Pre-post race (1st race)		0.0640	
Pre-post race (2nd race)		0.4206	
Panax ginseng			
Time (Pre – post race)	0.5383	0.4693	0.0189
10 km race (1st vs 2nd race)	0.54278	0.4674	0.0870

Multiple comparison			
Pre-race (1st race vs 2nd race)		0.6278	
Post-race (1st race vs 2nd race)		0.3959	
Pre-post race (1st race)		0.4107	
Pre-post race (2nd race)		0.8411	
**G. Non-esterified fatty acids**			
Before 10 km race			
Time (1st race vs 2nd race)	0.4456	0.5103	0.0169
Treatment (Placebo vs PG)	3.294	0.0811	0.2035
Multiple comparison			
Placebo (1st race vs 2nd race)		0.3026	
Panax ginseng (1st race vs 2nd race)		0.9470	
First race (Placebo vs PG)		0.0597	
Second race (Placebo vs PG)		0.3018	
After 10 km race			
Time (1st race vs 2nd race)	0.2038	0.6550	0.0070
Treatment (Placebo vs PG)	1.899	0.1787	0.0839
Multiple comparison			
Placebo (1st race vs 2nd race)		0.9898	
Panax ginseng (1st race vs 2nd race)		0.5267	
First race (Placebo vs PG)		0.1806	
Second race (Placebo vs PG)		0.4568	
Placebo			
Time (Pre – post race)	0.2287	0.6362	0.0081
10 km race (1st vs 2nd race)	0.3997	0.5324	0.0435
Multiple comparison			
Pre-race (1st race vs 2nd race)		0.4448	
Post-race (1st race vs 2nd race)		0.7398	
Pre-post race (1st race)		0.5188	
Pre-post race (2nd race)		0.9819	
Panax ginseng			
Time (Pre – post race)	0.0151	0.9032	0.0006
10 km race (1st vs 2nd race)	0.2743	0.6053	0.0147
Multiple comparison			
Pre-race (1st race vs 2nd race)		0.9453	
Post-race (1st race vs 2nd race)		0.4754	
Pre-post race (1st race)		0.6666	
Pre-post race (2nd race)		0.7954	
**H. Lactate**			
Before 10 km race			
Time (1st race vs 2nd race)	3.451	0.0746	0.1172
Treatment (Placebo vs PG)	0.1673	0.6859	0.0093
Multiple comparison			
Placebo (1st race vs 2nd race)		0.1860	
Panax ginseng (1st race vs 2nd race)		0.2142	
First race (Placebo vs PG)		0.7573	
Second race (Placebo vs PG)		0.7512	
After 10 km race			
Time (1st race vs 2nd race)	1.278	0.2685	0.0469

Treatment (Placebo vs PG)	10.12	0.0038	0.5482
Multiple comparison			
Placebo (1st race vs 2nd race)		0.8209	
Panax ginseng (1st race vs 2nd race)		0.1944	
First race (Placebo vs PG)		0.0025	
Second race (Placebo vs PG)		0.0218	
Placebo			
Time (Pre – post race)	45.57	<0.0001	0.6194
10 km race (1st vs 2nd race)	0.7375	0.3977	0.0185
Multiple comparison			
Pre-race (1st race vs 2nd race)		0.4623	
Post-race (1st race vs 2nd race)		0.7139	
Pre-post race (1st race)		<0.0001	
Pre-post race (2nd race)		<0.0001	
Panax ginseng			
Time (Pre – post race)	51.54	<0.0001	0.6823
10 km race (1st vs 2nd race)	0.7757	0.3872	0.0336
Multiple comparison			
Pre-race (1st race vs 2nd race)		0.7582	
Post-race (1st race vs 2nd race)		0.3428	
Pre-post race (1st race)		<0.0001	
Pre-post race (2nd race)		<0.0001	
**I. IL-1Ra**			
Before 10 km race			
Time (1st race vs 2nd race)	0.1260	0.7252	0.0043
Treatment (Placebo vs PG)	0.1151	0.7369	0.0152
Multiple comparison			
Placebo (1st race vs 2nd race)		0.4755	
Panax ginseng (1st race vs 2nd race)		0.2422	
First race (Placebo vs PG)		0.3623	
Second race (Placebo vs PG)		0.7552	
After 10 km race			
Time (1st race vs 2nd race)	0.0133	0.9087	0.0005
Treatment (Placebo vs PG)	0.3404	0.5641	0.0968
Multiple comparison			
Placebo (1st race vs 2nd race)		0.7556	
Panax ginseng (1st race vs 2nd race)		0.6452	
First race (Placebo vs PG)		0.4698	
Second race (Placebo vs PG)		0.7052	
Placebo			
Time (Pre – post race)	0.1504	0.7009	0.0050
10 km race (1st vs 2nd race)	0.3722	0.5464	0.0296
Multiple comparison			
Pre-race (1st race vs 2nd race)		0.4483	
Post-race (1st race vs 2nd race)		0.7915	
Pre-post race (1st race)		0.5521	
Pre-post race (2nd race)		0.9581	
Panax ginseng			
Time (Pre – post race)	1.221	0.2786	0.0418

10 km race (1st vs 2nd race)	0.1358	0.7153	0.0506
Multiple comparison			
Pre-race (1st race vs 2nd race)		0.5928	
Post-race (1st race vs 2nd race)		0.8674	
Pre-post race (1st race)		0.7453	
Pre-post race (2nd race)		0.2272	
**J. IL-6**			
Before 10 km race			
Time (1st race vs 2nd race)	9.398	0.0033	0.1394
Treatment (Placebo vs PG)	60.71	<0.0001	0.5115
Multiple comparison			
Placebo (1st race vs 2nd race)		<0.0001	
Panax ginseng (1st race vs 2nd race)		0.2183	
First race (Placebo vs PG)		<0.0001	
Second race (Placebo vs PG)		0.0422	
After 10 km race			
Time (1st race vs 2nd race)	10.19	0.0023	0.1494
Treatment (Placebo vs PG)	19.97	<0.0001	0.2561
Multiple comparison			
Placebo (1st race vs 2nd race)		0.0024	
Panax ginseng (1st race vs 2nd race)		0.1749	
First race (Placebo vs PG)		0.0002	
Second race (Placebo vs PG)		0.0252	
Placebo			
Time (Pre – post race)	29.64	<0.0001	0.3306
10 km race (1st vs 2nd race)	2.324	0.1327	0.0373
Multiple comparison			
Pre-race (1st race vs 2nd race)		0.8781	
Post-race (1st race vs 2nd race)		0.0498	
Pre-post race (1st race)		<0.0001	
Pre-post race (2nd race)		0.0048	
Panax ginseng			
Time (Pre – post race)	0.0464	0.8302	0.0008
10 km race (1st vs 2nd race)	27.49	<0.0001	0.3292
Multiple comparison			
Pre-race (1st race vs 2nd race)		0.1606	
Post-race (1st race vs 2nd race)		0.0897	
Pre-post race (1st race)		<0.0001	
Pre-post race (2nd race)		0.0373	
**K. IL-8**			
Before 10 km race			
Time (1st race vs 2nd race)	0.1777	0.6772	0.0077
Treatment (Placebo vs PG)	1.021	0.3227	0.0979
Multiple comparison			
Placebo (1st race vs 2nd race)		0.5618	
Panax ginseng (1st race vs 2nd race)		0.9672	
First race (Placebo vs PG)		0.5139	
Second race (Placebo vs PG)		0.3015	
After 10 km race			

Time (1st race vs 2nd race)	0.2971	0.5909	0.0128
Treatment (Placebo vs PG)	0.7773	0.3871	0.1461
Multiple comparison			
Placebo (1st race vs 2nd race)		0.9934	
Panax ginseng (1st race vs 2nd race)		0.4693	
First race (Placebo vs PG)		0.3077	
Second race (Placebo vs PG)		0.5649	
Placebo			
Time (Pre – post race)	4.478	0.0434	0.1379
10 km race (1st vs 2nd race)	0.5456	0.4663	0.0571
Multiple comparison			
Pre-race (1st race vs 2nd race)		0.3217	
Post-race (1st race vs 2nd race)		0.7765	
Pre-post race (1st race)		0.0543	
Pre-post race (2nd race)		0.3334	
Panax ginseng			
Time (Pre – post race)	7.444	0.0120	0.2445
10 km race (1st vs 2nd race)	0.3596	0.5546	0.0532
Multiple comparison			
Pre-race (1st race vs 2nd race)		0.8091	
Post-race (1st race vs 2nd race)		0.4177	
Pre-post race (1st race)		0.1566	
Pre-post race (2nd race)		0.0242	
**L. IL-10**			
Before 10 km race			
Time (1st race vs 2nd race)	0.5091	0.4812	0.0173
Treatment (Placebo vs PG)	0.4846	0.4919	0.0585
Multiple comparison			
Placebo (1st race vs 2nd race)		0.4002	
Panax ginseng (1st race vs 2nd race)		0.8690	
First race (Placebo vs PG)		0.4064	
Second race (Placebo vs PG)		0.6909	
After 10 km race			
Time (1st race vs 2nd race)	3.992	0.0552	0.1210
Treatment (Placebo vs PG)	1.100	0.3029	0.2108
Multiple comparison			
Placebo (1st race vs 2nd race)		0.7949	
Panax ginseng (1st race vs 2nd race)		0.0172	
First race (Placebo vs PG)		0.1248	
Second race (Placebo vs PG)		0.6864	
Placebo			
Time (Pre – post race)	27.77	<0.0001	0.4807
10 km race (1st vs 2nd race)	0.0013	0.9712	0.0001
Multiple comparison			
Pre-race (1st race vs 2nd race)		0.8381	
Post-race (1st race vs 2nd race)		0.8838	
Pre-post race (1st race)		0.0014	
Pre-post race (2nd race)		0.0005	
Panax ginseng			

Time (Pre – post race)	30.10	<0.0001	0.5181
10 km race (1st vs 2nd race)	2.197	0.1495	0.0966
Multiple comparison			
Pre-race (1st race vs 2nd race)		0.9480	
Post-race (1st race vs 2nd race)		0.0242	
Pre-post race (1st race)		0.0152	
Pre-post race (2nd race)		<0.0001	
**M. TNFα**			
Before 10 km race			
Time (1st race vs 2nd race)	0.5420	0.4687	0.0221
Treatment (Placebo vs PG)	1.537	0.2271	0.1835
Multiple comparison			
Placebo (1st race vs 2nd race)		0.6102	
Panax ginseng (1st race vs 2nd race)		0.6031	
First race (Placebo vs PG)		0.2666	
Second race (Placebo vs PG)		0.2929	
After 10 km race			
Time (1st race vs 2nd race)	1.641	0.2125	0.0640
Treatment (Placebo vs PG)	1.536	0.2272	0.3062
Multiple comparison			
Placebo (1st race vs 2nd race)		0.3314	
Panax ginseng (1st race vs 2nd race)		0.4106	
First race (Placebo vs PG)		0.2538	
Second race (Placebo vs PG)		0.2513	
Placebo			
Time (Pre – post race)	2.929	0.0977	0.0917
10 km race (1st vs 2nd race)	0.1840	0.6711	0.0216
Multiple comparison			
Pre-race (1st race vs 2nd race)		0.6296	
Post-race (1st race vs 2nd race)		0.7873	
Pre-post race (1st race)		0.1879	
Pre-post race (2nd race)		0.2945	
Panax ginseng			
Time (Pre – post race)	8.914	0.0068	0.2833
10 km race (1st vs 2nd race)	0.1013	0.7533	0.0163
Multiple comparison			
Pre-race (1st race vs 2nd race)		0.8942	
Post-race (1st race vs 2nd race)		0.6698	
Pre-post race (1st race)		0.0724	
Pre-post race (2nd race)		0.0290	

Table S3. Two-way ANOVA statistical analysis results for aerobic capacity, blood and perceived effort parameters of volunteer's sub-maximal aerobic test.

**Parameter**	**F**	**p-value**	**Partial eta squared**
**A. VO_2**			
Time (1st test vs 2nd test)	0.1121	0.7416	0.0062
Treatment (Placebo vs PG)	0.0898	0.2922	0.6638
Multiple comparison			
Placebo (1st test vs 2nd test)		0.0717	
Panax ginseng (1st test vs 2nd test)		0.0863	
First test (Placebo vs PG)		0.1326	
Second test (Placebo vs PG)		0.5544	
**B. VCO_2**			
Time (1st test vs 2nd test)	0.2284	0.6385	0.0125
Treatment (Placebo vs PG)	1.552	0.2288	0.5953
Multiple comparison			
Placebo (1st test vs 2nd test)		0.2188	
Panax ginseng (1st test vs 2nd test)		0.4315	
First test (Placebo vs PG)		0.1269	
Second test (Placebo vs PG)		0.3962	
**C. RER**			
Time (1st test vs 2nd test)	0.4600	0.5063	0.0249
Treatment (Placebo vs PG)	0.0803	0.7801	0.0066
Multiple comparison			
Placebo (1st test vs 2nd test)		0.5193	
Panax ginseng (1st test vs 2nd test)		0.7923	
First test (Placebo vs PG)		0.9973	
Second test (Placebo vs PG)		0.6663	
**D. VE**			
Time (1st test vs 2nd test)	0.4057	0.5322	0.0220
Treatment (Placebo vs PG)	0.0898	0.7678	0.0700
Multiple comparison			
Placebo (1st test vs 2nd test)		0.8897	
Panax ginseng (1st test vs 2nd test)		0.2536	
First test (Placebo vs PG)		0.9390	
Second test (Placebo vs PG)		0.6177	
**E. VE/VO_2**			
Time (1st test vs 2nd test)	0.0461	0.8324	0.0026
Treatment (Placebo vs PG)	0.1078	0.7465	0.0914
Multiple comparison			
Placebo (1st test vs 2nd test)		0.5957	
Panax ginseng (1st test vs 2nd test)		0.3301	
First test (Placebo vs PG)		0.9448	
Second test (Placebo vs PG)		0.5735	
**F. Heart rate**			
Time (1st test vs 2nd test)	0.5803	0.4561	0.0312
Treatment (Placebo vs PG)	0.0237	0.8792	0.0141
Multiple comparison			
Placebo (1st test vs 2nd test)		0.9735	

Panax ginseng (1st test vs 2nd test)		0.2285	
First test (Placebo vs PG)		0.7029	
Second test (Placebo vs PG)		0.9292	
**G. Borg's perceived exertion (CR-10)**			
Time (1st test vs 2nd test)	0.2483	0.6243	0.0136
Treatment (Placebo vs PG)	0.4303	0.5201	0.2565
Multiple comparison			
Placebo (1st test vs 2nd test)		>0.9999	
Panax ginseng (1st test vs 2nd test)		0.4410	
First test (Placebo vs PG)		0.4515	
Second test (Placebo vs PG)		0.6149	
**H. Lactate**			
Before Sub-maximal aerobic test			
Time (1st test vs 2nd test)	3.237	0.0888	0.1524
Treatment (Placebo vs PG)	0.0537	0.8194	0.0099
Multiple comparison			
Placebo (1st test vs 2nd test)		0.2090	
Panax ginseng (1st test vs 2nd test)		0.2277	
First test (Placebo vs PG)		0.9223	
Second test (Placebo vs PG)		0.7595	
After Sub-maximal aerobic test			
Time (1st test vs 2nd test)	0.6512	0.4302	0.0349
Treatment (Placebo vs PG)	0.0337	0.8565	0.0049
Multiple comparison			
Placebo (1st test vs 2nd test)		0.2913	
Panax ginseng (1st test vs 2nd test)		0.9564	
First test (Placebo vs PG)		0.7634	
Second test (Placebo vs PG)		0.5419	
Placebo			
Time (Pre – post test)	36.02	<0.0001	0.7201
Sub-maximal aerobic test (1st vs 2nd)	1.460	0.2470	0.1353
Multiple comparison			
Pre-test (1st vs 2nd)		0.4403	
Post-test (1st vs 2nd test)		0.2854	
Pre-post test (1st test)		0.0006	
Pre-post test (2nd test)		0.0011	
Panax ginseng			
Time (Pre – post test)	53.33	<0.0001	0.7080
Sub-maximal aerobic test (1st vs 2nd)	0.1106	0.7426	0.0132
Multiple comparison			
Pre-test (1st vs 2nd)		0.5436	
Post-test (1st vs 2nd test)		0.9640	
Pre-post test (1st test)		0.0001	
Pre-post test (2nd test)		<0.0001	
**I. Total lipids**			
Before Sub-maximal aerobic test			
Time (1st test vs 2nd test)	0.2086	0.6533	0.0115
Treatment (Placebo vs PG)	0.0016	0.9680	0.0005
Multiple comparison			

Placebo (1st test vs 2nd test)		0.0418	
Panax ginseng (1st test vs 2nd test)		0.0031	
First test (Placebo vs PG)		0.1378	
Second test (Placebo vs PG)		0.1568	
After Sub-maximal aerobic test			
Time (1st test vs 2nd test)	0.0764	0.7853	0.0042
Treatment (Placebo vs PG)	0.0022	0.9630	0.0003
Multiple comparison			
Placebo (1st test vs 2nd test)		0.5540	
Panax ginseng (1st test vs 2nd test)		0.7660	
First test (Placebo vs PG)		0.7656	
Second test (Placebo vs PG)		0.7055	
Placebo			
Time (Pre – post test)	3.742	0.0735	0.2109
Sub-maximal aerobic test (1st vs 2nd)	1.478	0.2442	0.1816
Multiple comparison			
Pre-test (1st vs 2nd)		0.1596	
Post-test (1st vs 2nd test)		0.5821	
Pre-post test (1st test)		0.0753	
Pre-post test (2nd test)		0.4287	
Panax ginseng			
Time (Pre – post test)	3.032	0.0956	0.1211
Sub-maximal aerobic test (1st vs 2nd)	1.174	0.2904	0.1253
Multiple comparison			
Pre-test (1st vs 2nd)		0.1070	
Post-test (1st vs 2nd test)		0.8394	
Pre-post test (1st test)		0.8028	
Pre-post test (2nd test)		0.0378	
**J. Phospholipids**			
Before Sub-maximal aerobic test			
Time (1st test vs 2nd test)	0.0216	0.8841	0.0006
Treatment (Placebo vs PG)	7.787	0.0084	0.1779
Multiple comparison			
Placebo (1st test vs 2nd test)		>0.9999	
Panax ginseng (1st test vs 2nd test)		0.8177	
First test (Placebo vs PG)		0.0697	
Second test (Placebo vs PG)		0.0450	
After Sub-maximal aerobic test			
Time (1st test vs 2nd test)	0.1557	0.6955	0.0043
Treatment (Placebo vs PG)	1.147	0.2913	0.0309
Multiple comparison			
Placebo (1st test vs 2nd test)		0.7181	
Panax ginseng (1st test vs 2nd test)		0.8595	
First test (Placebo vs PG)		0.5277	
Second test (Placebo vs PG)		0.3864	
Placebo			
Time (Pre – post test)	7.660	0.0099	0.2148
Sub-maximal aerobic test (1st vs 2nd)	0.0589	0.8099	0.0021
Multiple comparison			

Pre-test (1st vs 2nd)		>0.9999	
Post-test (1st vs 2nd test)		0.7339	
Pre-post test (1st test)		0.0850	
Pre-post test (2nd test)		0.0422	
Panax ginseng			
Time (Pre – post test)	57.59	<0.0001	0.5669
Sub-maximal aerobic test (1st vs 2nd)	0.0177	0.8947	0.0004
Multiple comparison			
Pre-test (1st vs 2nd)		0.8515	
Post-test (1st vs 2nd test)		0.7083	
Pre-post test (1st test)		<0.0001	
Pre-post test (2nd test)		<0.0001	
**K. Total cholesterol**			
Before Sub-maximal aerobic test			
Time (1st test vs 2nd test)	0.1730	0.6824	0.0095
Treatment (Placebo vs PG)	1.137	0.3005	0.3183
Multiple comparison			
Placebo (1st test vs 2nd test)		0.5928	
Panax ginseng (1st test vs 2nd test)		0.9928	
First test (Placebo vs PG)		0.3993	
Second test (Placebo vs PG)		0.2585	
After Sub-maximal aerobic test			
Time (1st test vs 2nd test)	3.315	0.0853	0.1555
Treatment (Placebo vs PG)	1.374	0.2563	0.1687
Multiple comparison			
Placebo (1st test vs 2nd test)		0.0720	
Panax ginseng (1st test vs 2nd test)		0.5974	
First test (Placebo vs PG)		0.6893	
Second test (Placebo vs PG)		0.1193	
Placebo			
Time (Pre – post test)	1.406	0.2555	0.0913
Sub-maximal aerobic test (1st vs 2nd)	0.5902	0.4551	0.1812
Multiple comparison			
Pre-test (1st vs 2nd)		0.8367	
Post-test (1st vs 2nd test)		0.2401	
Pre-post test (1st test)		0.9699	
Pre-post test (2nd test)		0.1083	
Panax ginseng			
Time (Pre – post test)	2.863	0.1157	0.1087
Sub-maximal aerobic test (1st vs 2nd)	0.0778	0.7829	0.0130
Multiple comparison			
Pre-test (1st vs 2nd)		0.9959	
Post-test (1st vs 2nd test)		0.6192	
Pre-post test (1st test)		0.4500	
Pre-post test (2nd test)		0.1361	
**L. Triacylglycerides**			
Before Sub-maximal aerobic test			
Time (1st test vs 2nd test)	0.5781	0.4581	0.0349
Treatment (Placebo vs PG)	1.777	0.2012	0.0793

Multiple comparison			
Placebo (1st test vs 2nd test)		0.9154	
Panax ginseng (1st test vs 2nd test)		0.2254	
First test (Placebo vs PG)		0.1257	
Second test (Placebo vs PG)		0.8507	
After Sub-maximal aerobic test			
Time (1st test vs 2nd test)	0.6416	0.4349	0.0386
Treatment (Placebo vs PG)	2.714	0.1190	0.1349
Multiple comparison			
Placebo (1st test vs 2nd test)		0.8960	
Panax ginseng (1st test vs 2nd test)		0.3080	
First test (Placebo vs PG)		0.1250	
Second test (Placebo vs PG)		0.4860	
Placebo			
Time (Pre – post test)	4.512	0.0520	0.2437
Sub-maximal aerobic test (1st vs 2nd)	0.0006	0.9805	0.0003
Multiple comparison			
Pre-test (1st vs 2nd)		0.8327	
Post-test (1st vs 2nd test)		0.7971	
Pre-post test (1st test)		0.0701	
Pre-post test (2nd test)		0.3146	
Panax ginseng			
Time (Pre – post test)	13.56	0.0015	0.4041
Sub-maximal aerobic test (1st vs 2nd)	0.4426	0.5135	0.3372
Multiple comparison			
Pre-test (1st vs 2nd)		0.3835	
Post-test (1st vs 2nd test)		0.6758	
Pre-post test (1st test)		0.0990	
Pre-post test (2nd test)		0.0019	
**M. Non-esterified fatty acids**			
Before Sub-maximal aerobic test			
Time (1st test vs 2nd test)	3.424	0.0725	0.0869
Treatment (Placebo vs PG)	14.40	0.0005	0.2858
Multiple comparison			
Placebo (1st test vs 2nd test)		0.0364	
Panax ginseng (1st test vs 2nd test)		0.9938	
First test (Placebo vs PG)		0.3704	
Second test (Placebo vs PG)		0.0005	
After Sub-maximal aerobic test			
Time (1st test vs 2nd test)	0.0297	0.8640	0.0008
Treatment (Placebo vs PG)	12.50	0.0011	0.2577
Multiple comparison			
Placebo (1st test vs 2nd test)		0.7404	
Panax ginseng (1st test vs 2nd test)		0.4998	
First test (Placebo vs PG)		0.0517	
Second test (Placebo vs PG)		0.0050	
Placebo			
Time (Pre – post test)	1.527	0.2269	0.0517
Sub-maximal aerobic test (1st vs 2nd)	1.644	0.2103	0.0555

Multiple comparison			
Pre-test (1st vs 2nd)		0.0540	
Post-test (1st vs 2nd test)		0.8446	
Pre-post test (1st test)		0.8192	
Pre-post test (2nd test)		0.0578	
Panax ginseng			
Time (Pre – post test)	94.94	<0.0001	0.6833
Sub-maximal aerobic test (1st vs 2nd)	0.2700	0.6059	0.0061
Multiple comparison			
Pre-test (1st vs 2nd)		0.8551	
Post-test (1st vs 2nd test)		0.3633	
Pre-post test (1st test)		<0.0001	
Pre-post test (2nd test)		<0.0001	
**N. IL-1ra**			
Before Sub-maximal aerobic test			
Time (1st test vs 2nd test)	1.803	0.2042	0.1307
Treatment (Placebo vs PG)	4.271	0.0611	0.7274
Multiple comparison			
Placebo (1st test vs 2nd test)		0.1228	
Panax ginseng (1st test vs 2nd test)		0.8954	
First test (Placebo vs PG)		0.1364	
Second test (Placebo vs PG)		0.0278	
After Sub-maximal aerobic test			
Time (1st test vs 2nd test)	0.1802	0.6787	0.0148
Treatment (Placebo vs PG)	4.665	0.0517	0.2952
Multiple comparison			
Placebo (1st test vs 2nd test)		0.5179	
Panax ginseng (1st test vs 2nd test)		0.9059	
First test (Placebo vs PG)		0.2610	
Second test (Placebo vs PG)		0.0618	
Placebo			
Time (Pre – post test)	2.816	0.1243	0.2197
Sub-maximal aerobic test (1st vs 2nd)	0.4197	0.5317	0.0887
Multiple comparison			
Pre-test (1st vs 2nd)		0.6318	
Post-test (1st vs 2nd test)		0.5577	
Pre-post test (1st test)		0.2905	
Pre-post test (2nd test)		0.2373	
Panax ginseng			
Time (Pre – post test)	9.211	0.0075	0.3514
Sub-maximal aerobic test (1st vs 2nd)	0.3012	0.5903	0.0677
Multiple comparison			
Pre-test (1st vs 2nd)		0.6957	
Post-test (1st vs 2nd test)		0.5594	
Pre-post test (1st test)		0.0302	
Pre-post test (2nd test)		0.0692	
**O. IL-6**			
Before Sub-maximal aerobic test			
Time (1st test vs 2nd test)	1.004	0.3399	0.0913

Treatment (Placebo vs PG)	0.3070	0.5917	0.1805
Multiple comparison			
Placebo (1st test vs 2nd test)		0.8386	
Panax ginseng (1st test vs 2nd test)		0.2548	
First test (Placebo vs PG)		0.7884	
Second test (Placebo vs PG)		0.4525	
After Sub-maximal aerobic test			
Time (1st test vs 2nd test)	1.269	0.2863	0.1126
Treatment (Placebo vs PG)	1.969	0.1908	0.3098
Multiple comparison			
Placebo (1st test vs 2nd test)		0.4270	
Panax ginseng (1st test vs 2nd test)		0.4618	
First test (Placebo vs PG)		0.2463	
Second test (Placebo vs PG)		0.2656	
Placebo			
Time (Pre – post test)	2.724	0.1298	0.2141
Sub-maximal aerobic test (1st vs 2nd)	0.2306	0.6414	0.0503
Multiple comparison			
Pre-test (1st vs 2nd)		0.9314	
Post-test (1st vs 2nd test)		0.4832	
Pre-post test (1st test)		0.1476	
Pre-post test (2nd test)		0.4622	
Panax ginseng			
Time (Pre – post test)	0.6389	0.4385	0.0469
Sub-maximal aerobic test (1st vs 2nd)	0.9457	0.3486	0.6018
Multiple comparison			
Pre-test (1st vs 2nd)		0.4449	
Post-test (1st vs 2nd test)		0.2713	
Pre-post test (1st test)		0.2250	
Pre-post test (2nd test)		0.9935	
**P. IL-8**			
Before Sub-maximal aerobic test			
Time (1st test vs 2nd test)	0.8066	0.3833	0.0510
Treatment (Placebo vs PG)	0.0063	0.9378	0.0031
Multiple comparison			
Placebo (1st test vs 2nd test)		0.4535	
Panax ginseng (1st test vs 2nd test)		0.6453	
First test (Placebo vs PG)		0.8489	
Second test (Placebo vs PG)		0.9658	
After Sub-maximal aerobic test			
Time (1st test vs 2nd test)	0.0462	0.8328	0.0031
Treatment (Placebo vs PG)	0.7412	0.4028	0.2442
Multiple comparison			
Placebo (1st test vs 2nd test)		0.8307	
Panax ginseng (1st test vs 2nd test)		0.9475	
First test (Placebo vs PG)		0.4576	
Second test (Placebo vs PG)		0.4015	
Placebo			
Time (Pre – post test)	3.950	0.0723	0.2642

Sub-maximal aerobic test (1st vs 2nd)	0.1300	0.7252	0.0647
Multiple comparison			
Pre-test (1st vs 2nd)		0.7136	
Post-test (1st vs 2nd test)		0.7710	
Pre-post test (1st test)		0.1825	
Pre-post test (2nd test)		0.1924	
Panax ginseng			
Time (Pre – post test)	3.030	0.0971	0.1316
Sub-maximal aerobic test (1st vs 2nd)	0.0083	0.9285	0.0025
Multiple comparison			
Pre-test (1st vs 2nd)		0.8342	
Post-test (1st vs 2nd test)		0.9666	
Pre-post test (1st test)		0.3327	
Pre-post test (2nd test)		0.1574	
**Q. IL-10**			
Before Sub-maximal aerobic test			
Time (1st test vs 2nd test)	0.0167	0.8991	0.0013
Treatment (Placebo vs PG)	0.2379	0.6339	0.0459
Multiple comparison			
Placebo (1st test vs 2nd test)		0.4236	
Panax ginseng (1st test vs 2nd test)		0.4339	
First test (Placebo vs PG)		0.3176	
Second test (Placebo vs PG)		0.8518	
After Sub-maximal aerobic test			
Time (1st test vs 2nd test)	0.1180	0.7367	0.0090
Treatment (Placebo vs PG)	0.1434	0.7111	0.0274
Multiple comparison			
Placebo (1st test vs 2nd test)		0.6454	
Panax ginseng (1st test vs 2nd test)		0.9736	
First test (Placebo vs PG)		0.9084	
Second test (Placebo vs PG)		0.6035	
Placebo			
Time (Pre – post test)	5.516	0.0443	0.3191
Sub-maximal aerobic test (1st vs 2nd)	1.207	0.2954	0.1551
Multiple comparison			
Pre-test (1st vs 2nd)		0.1189	
Post-test (1st vs 2nd test)		0.1546	
Pre-post test (1st test)		0.3854	
Pre-post test (2nd test)		0.4031	
Panax ginseng			
Time (Pre – post test)	7.370	0.0147	0.3024
Sub-maximal aerobic test (1st vs 2nd)	0.0057	0.9408	0.0064
Multiple comparison			
Pre-test (1st vs 2nd)		0.8591	
Post-test (1st vs 2nd test)		0.7465	
Pre-post test (1st test)		0.2687	
Pre-post test (2nd test)		0.0166	
**R. TNFα**			
Before Sub-maximal aerobic test			

Time (1st test vs 2nd test)	0.1616	0.6937	0.0114
Treatment (Placebo vs PG)	0.0262	0.8737	0.0103
Multiple comparison			
Placebo (1st test vs 2nd test)		0.8788	
Panax ginseng (1st test vs 2nd test)		0.6554	
First test (Placebo vs PG)		0.8350	
Second test (Placebo vs PG)		0.9306	
After Sub-maximal aerobic test			
Time (1st test vs 2nd test)	1.549	0.2337	0.0996
Treatment (Placebo vs PG)	1.979	0.1813	0.2146
Multiple comparison			
Placebo (1st test vs 2nd test)		0.0888	
Panax ginseng (1st test vs 2nd test)		0.7474	
First test (Placebo vs PG)		0.0445	
Second test (Placebo vs PG)		0.8580	
Placebo			
Time (Pre – post test)	6.264	0.0294	0.3629
Sub-maximal aerobic test (1st vs 2nd)	1.896	0.1959	0.2297
Multiple comparison			
Pre-test (1st vs 2nd)		0.9286	
Post-test (1st vs 2nd test)		0.0476	
Pre-post test (1st test)		0.0108	
Pre-post test (2nd test)		0.5779	
Panax ginseng			
Time (Pre – post test)	3.154	0.0917	0.1424
Sub-maximal aerobic test (1st vs 2nd)	0.0202	0.8885	0.0169
Multiple comparison			
Pre-test (1st vs 2nd)		0.9147	
Post-test (1st vs 2nd test)		0.8675	
Pre-post test (1st test)		0.2464	
Pre-post test (2nd test)		0.2048	
